# The Gut Microbiota and Inflammatory Factors in Pediatric Appendicitis

**DOI:** 10.1155/2022/1059445

**Published:** 2022-07-07

**Authors:** Yuewei Bi, Qianyu Yang, Jiao Li, Xufeng Zhao, Beilei Yan, Xuan Li, Hualei Cui

**Affiliations:** ^1^Department of the Graduate School, Tianjin Medical University, Tianjin, China; ^2^Department of Internal Medicine, Tianjin Children's Hospital (Children's Hospital of Tianjin University), Tianjin, China; ^3^Department of General Surgery, Tianjin Children's Hospital (Children's Hospital of Tianjin University), Tianjin, China

## Abstract

**Background:**

The study analyzed gut microflora's composition and investigated the associations between the associations between gut dysbiosis and inflammatory indicators in pediatric patients with acute appendicitis.

**Methods:**

High-throughput sequencing and bioinformatics analysis were used to investigate the composition and diversity of gut microflora in 20 pediatric patients with acute appendicitis and 11 healthy children. Endpoints measured were operational taxonomic units (OTU) of gut microflora. The OTU and its abundance analysis, sample diversity analysis, principal component analysis of samples, differential analysis, and analysis of biomarkers were performed.

**Results:**

Overall fecal microbial richness and diversity were similar in patients and controls. Yet richness within the group of Bilophila, Eggerthella, Clostridium, Parvimonas, Megasphaera, Atopobium, Phascolarctobacterium, Adlercreutzia, Barnesiella, Klebsiella, Enterococcus, and Prevotella genera was higher in patients. Adlercreutzia was significantly positively correlated with IL-10, while the three other genera, comprising Klebsiella, Adlercreutzia, and Prevotella, were positively correlated with B cells level.

**Conclusion:**

Gut microbiome components are significantly different in pediatric patients with acute appendicitis and healthy children. The differential abundance of some genera is correlated with the production of inflammatory markers in appendicitis.

## 1. Introduction

Acute appendicitis (AA) is one of the most common pediatric surgical diseases worldwide with cumulative risk estimated between 6% and 17% [1–3]. The AA attack is more common among children from 10 to 19 years old [4]. Antibiotics have still been the appropriate first-line therapy for patients, with uncomplicated appendicitis compared to surgery [5, 6]. The occurrence of appendicitis was correlated to the metabolic disorder induced by abnormal bacterial colonization, suggesting that bacterial infection has been involved in the pathophysiology [1, 6].

Appendicitis occurs in clusters and varies seasonally, which may be a starting event in the pathogenesis of appendicitis [7–9]. The altered bowel behavior, microbiological infection, and lifestyle have been demonstrated as illness risk factors. For example, low-fiber diets and higher sugar intake promoted AA progression [10–12]. Breastfeeding drives favorable gut microflora colonization, which can protect against appendicitis [13]. The gut microbial communities of subjects with appendicitis were different from healthy people with antibiotic use [7, 14]. However, the effects of the gut microflora on appendicitis formation and progression without antibiotic use is less known.

In this study, 16S ribosomal DNA (rDNA) gene sequencing was applied to explore the constituents of gut microflora of patients without antibiotic use and investigate the associations between gut dysbiosis and inflammatory mediators in pediatric AA patients, which help prevent the potential AA progression and improve the prognosis.

## 2. Methods

### 2.1. Sample Collection and Processing

The samples were collected at Tianjin Children's Hospital between June 2020 and December 2020. Fecal and blood samples were collected from 20 pediatric AA patients after an appendectomy, called the P group. The stool specimens were collected from 11 normal children in the Y group control. After admission, fecal samples were sampled and immediately stored in the refrigerator at -80°C. This prospective study has been approved by the ethics board of Tianjin Children's Hospital (L2020-44) and gained informed consent from all children's parents or legal guardians.

Inclusion criteria were as follows: (1) patients were conscious and had stable vital signs; (2) boys and girls aged <18 years; (3) those with a pathological diagnosis of appendicitis after an appendectomy; (4) those without a special diet (e.g., vegetarian); and (5) those with no other disease or history of surgical treatment.

Exclusion criteria were as follows: (1) antibiotic, probiotic, or glucocorticoid use within four weeks before sampling; (2) the presence of congenital diseases; (3) the presence of other infectious diseases; and (4) receiving any interventions or operation treatments.

### 2.2. Extraction of Total DNA and Amplification by Polymerase Chain Reaction (PCR)

According to the manufacturer's instruction, genomic DNA was extracted using a DNA extraction kit (OMEGA-soil DNA Kit, Omega Bio-Tek, USA). DNA concentrations and purity were measured using an ultra-microvolume spectrophotometer (NanoDrop2000, Thermo Fisher). The bands were resolved on 1% agarose gel, and gene integrity was confirmed after that PCR was performed.

The primer sequences of the 16S rDNA V3–V4 variable region were as follows: F: 5′-CCTAYGGGRBGCASCAG-3′ and R: 5′-GGACTACHVGGGTWTCTAAT-3′.

### 2.3. High-Throughput Sequencing and Bioinformatics Analysis

PCR products of the 16S rDNA V3–V4 variable region were sequenced using a NovaSeq 6000 platform (American Illumina Company, San Diego). The connected tags were clustered as an operational taxonomic unit (OTU) with the Quantitative Insights into Microbial Ecology version 2 (QIIME2) software suite (QIIME 2 Development Team; https://qiime2.org/). A supervised comparison of the microflora was conducted through linear discriminant (LDA) and effect size (LEfSe) to identify the presence and impact of region-specific OTUs in different groups. The OTU and its abundance analysis, sample diversity analysis, principal component analysis of samples, differential analysis, and analysis of biomarkers were performed using bioinformatics analysis by QIIME2 software.

### 2.4. Flow Cytometry

Flow cytometry was performed immediately on fresh blood aspirates in all participants. The samples were collected in EDTA or heparin anticoagulant and routinely processed using a red cell lysis method. Cell suspensions were incubated with seven monoclonal antibodies (Becton Dickinson, San Jose, CA, USA): CD3 FITC, CD4 PE-Cy7, CD8 APC-Cy7, CD16 PE, CD56 PE, CD45 PerCP-Cy5.5, and CD19 APC. The cells were washed and resuspended in fluorescence-activated cell sorting (FACS) buffer before FACS analysis on a FACSCanto-II (Becton Dickinson).

### 2.5. Cytometric Bead Array Immunoassay

Blood sample concentration of IL-2, IL-4, IL-6, IL-10, IL-17, TNF, and IFN was simultaneously measured by a CBA kit (Becton Dickinson, San Diego, CA, USA). Briefly, for each serum sample and cytokine standard mixture, 25 *μ*l of sample or standard was added to a mixture of 25 *μ*l each of capture Abbead reagent and detector Ab-PE reagent. The mixture was subsequently incubated for 2.5 h at room temperature and washed to remove unbound detector Ab-PE reagent before data acquisition using flow cytometry. Two-color flow cytometric analysis was performed using FACSCanto-II (Becton Dickinson). Data were acquired and analyzed, and standard curves were plotted both using Becton Dickinson FCAP (3.0.1) software. Cytokine concentrations were determined from these standard curves.

### 2.6. Statistical Analysis

Normality of the distributions was evaluated. Continuous data with a normal distribution are expressed as means ± standard deviation (SD), and nonnormally distributed variables are expressed as medians ± interquartile ranges (IQRs; 25th and 75th percentiles). For normally distributed quantitative data (such as IL-17, CD3+ T cell, CD4+ T cell, CD8+ T cell, and CD4/CD8 ratio), an independent *t*-test was used to compare the differences. For nonnormally distributed quantitative data (age, IL-2, IL-4, IL-6, IL-10, TNF, IFN, NK cell, and B cell), the Mann-Whitney *U* test was used to detect statistical significance. Categorical data were compared between the groups using the *χ*^2^ test. Clinical data were analyzed using SPSS 21.0 version. A *P* < 0.05 was considered statistically significant.

## 3. Results

### 3.1. Baseline Clinical Characteristics

Thirty-one children were enrolled in our prospective study demonstrating that IL-10 expression was higher and NK cells and B cell infiltration increased in the P group (Table 1 and Tab. S1). There was no significant difference in other cytokines, such as IL-2, IL-4, IL-6, TNF, IFN, and IL-17, and cell infiltration, such as CD3+ T cells, CD4+ T cells, and the CD4+/CD8+ T cell ratio between the two groups.

### 3.2. Operational Taxonomic Unit Clustering and Annotation

The proportion of annotations at different levels was shown in Tab. S2. The species were mainly distributed into the following eight types of phyla: *Firmicutes*, *Bacteroidetes*, *Actinobacteria*, *Proteobacteria*, *Verrucomicrobia*, *Synergistetes*, *Euryarchaeota*, and *Fusobacteria* (Table 2 and Fig. S1). There was a 2.1% decrease in the abundance of *Firmicutes*, 1.1% in the abundance of *Bacteroidetes*, 1.9% in the abundance of *Actinobacteria*, and 4.2% increase in the abundance of *Proteobacteria* in the P group.

Microflora in the P group mainly included *Bacteroides* (25.8%), *Faecalibacterium* (13.8%), and *Bifidobacterium* (7.2%), while microflora in the Y group mainly included *Bacteroides* (30.0%), *Faecalibacterium* (17.3%), and *Bifidobacterium* (9.2%) (Fig. S2).

### 3.3. Alteration in Pediatric Patients from Health to Acute Appendicitis

**Figure 1:**
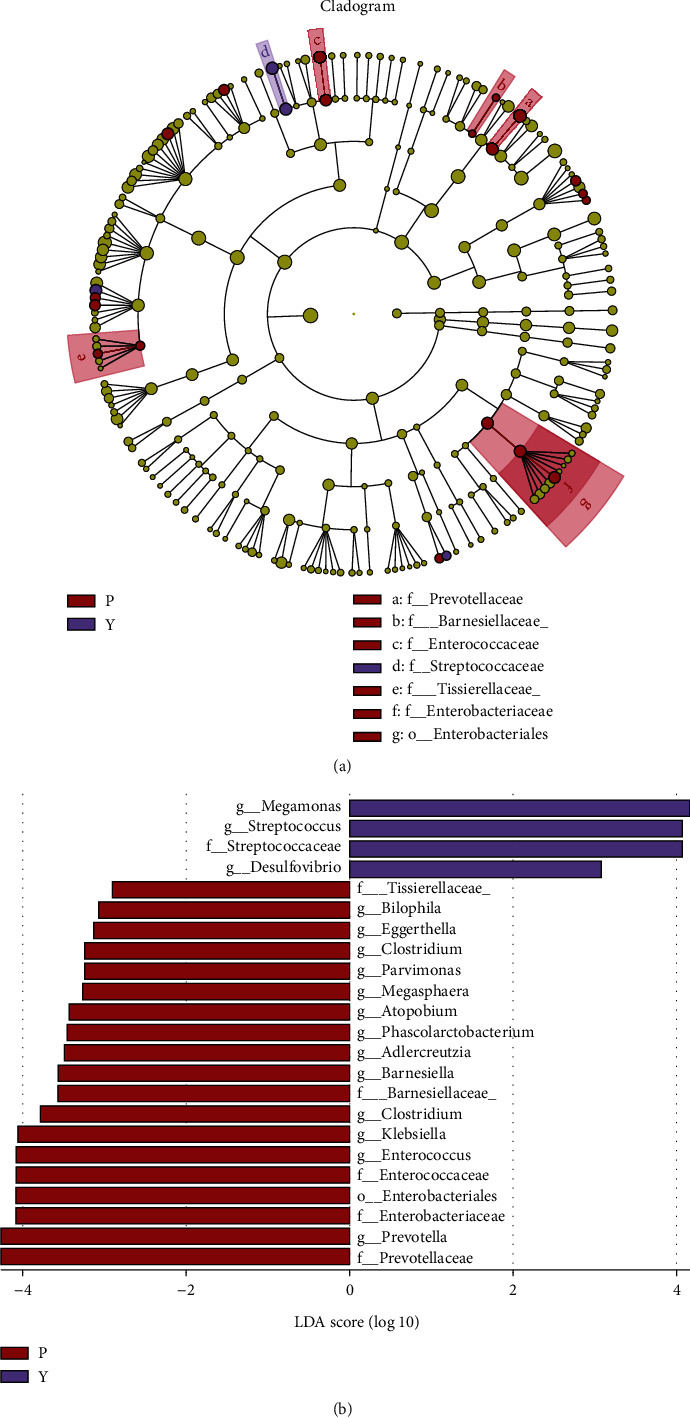
Taxonomic differences in the fecal microbiota exhibited by pediatric patients with acute appendicitis (P group) and healthy children group (Y group). A linear discriminant analysis (LDA; (log10) > 2) and effect size (LEfSe) analysis revealed significant differences in the fecal microbiota exhibited by P group (red, negative score) and Y group (purple, positive score). The different color nodes represent microbiota communities that are significantly enriched in the corresponding groups and significantly influence the differences between the groups; the pale yellow nodes indicate that the microbial groups either have no significant effect on the different groups or have no significant effect on the differences between groups. Linear discriminant analysis (LDA) integrated with effect size (LEfSe). (a) Cladogram indicating the phylogenetic distribution of microbiota correlated with the P or Y groups. (b) The differences in abundance between the P and Y groups.

**Figure 2:**
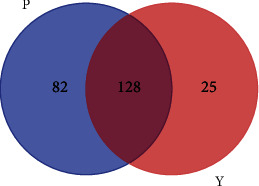
Venn diagram analysis. Different colors represent different groups, overlapping parts represent genera that are common both two groups, parts that do not overlap represent genera that are specific to the group, and numbers indicate the number of corresponding genera. Group P: pediatric acute appendicitis group; Group Y: healthy children group.

The logarithmic LDA score demonstrated a remarkable difference in the fecal microflora between the two groups. The relative abundances of the genera *Bilophila*, *Eggerthella*, *Clostridium*, *Parvimonas*, *Megasphaera*, *Atopobium*, *Phascolarctobacterium*, *Adlercreutzia*, *Barnesiella*, *Klebsiella*, *Enterococcus*, and *Prevotella* were higher in the P group (LDA score (log10) > 2). In contrast, *Megamonas*, *Streptococcus*, and *Desulfovibrio* were mainly enriched in the Y group (Figure 1). However, using the Venn diagram, 128 core genera were shared between two groups, including *Bilophila*, *Streptococcus*, and *Desulfovibrio* (Figure 2).

### 3.4. Phylogenetic Tree Analysis

**Figure 3:**
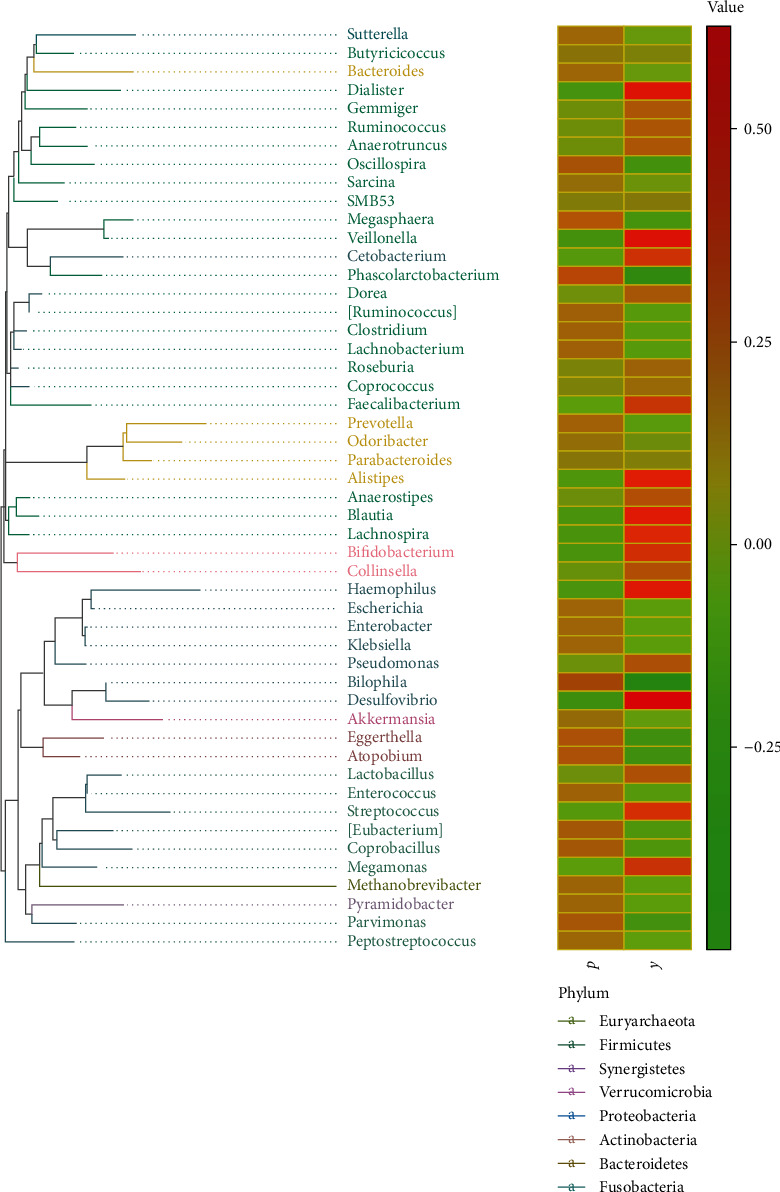
Phylogenetic tree analysis. On the left is the phylogenetic tree. The different colors of the branches of the phylogenetic tree represent different phyla. The length of the branch is the evolutionary distance between two species, namely, the degree of species difference. The right histogram shows the average number of reads belonging to different genus in each treatment. Group P: pediatric acute appendicitis group; Group Y: healthy children group.

The phylogenetic tree on the genus bar revealed the phylogenetic order of the classification in the evolution process from the perspective of molecular evolution. In our results, the phylogenetic tree showed the top 50 species in the total classification level. Compared with the Y group, the P group showed a decreased number of reads of *Desulfovibrio* (*Proteobacteria*), *Haemophilus* (*Proteobacteria*), *Blautia* (*Firmicutes*), *Veillonella* (*Firmicutes*), and *Dialister* (*Firmicutes*) (Figure 3). On the contrary, the number of reads of *Bilophila* (*Proteobacteria*) increased. Compared with the Y group, the P group had enriched *Bilophila* (phylum: *Proteobacteria*) and worsened *Proteobacteria* and the *Firmicutes* phyla.

### 3.5. The Diagnostic Value of Gut Biomarkers in Pediatric Acute Appendicitis

**Figure 4:**
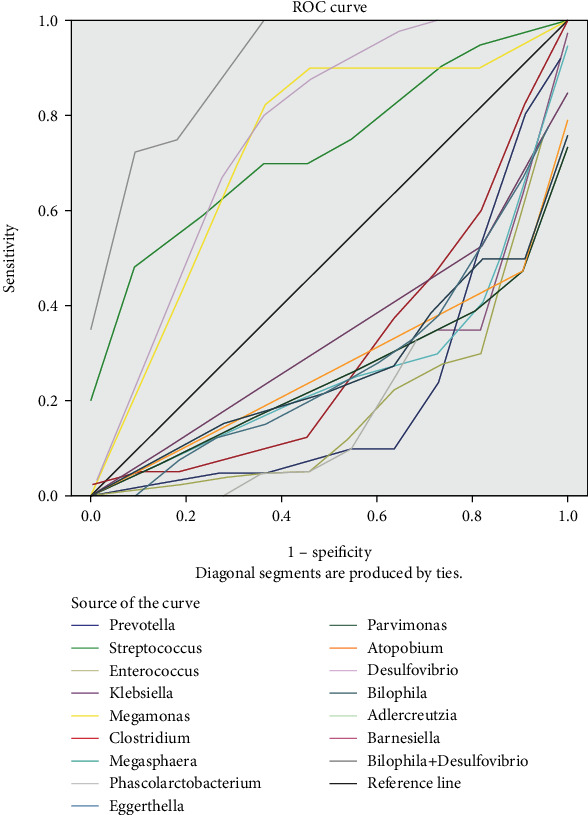
Receiver operating characteristic curve (ROC) analysis of the sensitivity and specificity of the differentially abundant genera as diagnostic factors for pediatric acute appendicitis.

A receiver operating characteristic (ROC) curve analysis was used to compare the diagnostic value of gut biomarkers (Figure 4). The area under the curves (AUCs) of the genera closely related to pediatric AA, for instance, *Desulfovibrio*, *Streptococcus*, and *Megamonas* were 0.752, 0.741, and 0.725, respectively. The joint AUC of the combination of *Desulfovibrio* and *Bilophila* was 0.927, suggesting the high diagnostic value of gut biomarkers in pediatric acute appendicitis.

### 3.6. The Diversity of the Gut Microflora

**Figure 5:**
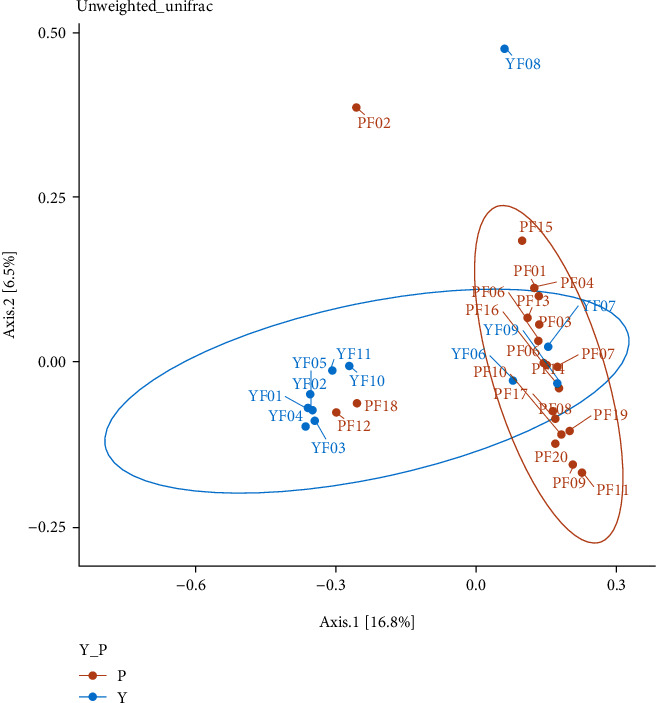
Principal coordinates analysis (PCoA) of unweighted UniFrac distances of 16S rRNA genes. Samples from acute appendicitis subjects (P group) were separated from those obtained from healthy children (Y group) (PERMANOVA, *P* < 0.05). Group P: pediatric acute appendicitis group (yellow dots); Group Y: healthy children group (blue dots), where dots represent individual samples.

Utilizing alpha diversity of the gut microflora, there was no significant difference between the two groups (Tab. S3 and Fig. S3). However, the PCoA showed that the stool microflora in the P group clustered far separately from those in the Y group (Figure 5). The permutational multivariate analysis also revealed significant differences in the constitution of the gut microflora between the two groups (*P* < 0.05).

### 3.7. Analysis of the Interaction Networks among the Genera

The correlation analysis was utilized based on relative abundances among genera, and the interaction networks in the P group are presented in Supplementary Figure S5. Among the genera of significantly different microflora, *Klebsiella*, *Enterococcus*, *Clostridium*, and *Atopobium* had a higher connection with the other genera in the samples. *Klebsiella* was positively associated with *Enterococcus* and *Eggerthella* without comma while negatively correlated with *Streptococcus*, *Akkermansia*, *Blautia*, and *Dorea*. *Enterococcus* was positively correlated with *Klebsiella*, *Clostridium*, *Eggerthella*, *Coprobacillus*, and *Atopobium* while negatively correlated with *Megamonas*. *Clostridium* was positively correlated with *Enterococcus*, *Eggerthella*, *Pseudomonas*, *Parvimonas*, and *Atopobium*. *Atopobium* was positively correlated with *Enterococcus*, *Clostridium*, and *Eggerthella* while negatively correlated with *Megamonas*, *Enterobacter*, *Sarcina*, and *Anaerostipes*.

### 3.8. Potential Connections between Fecal Microflora and Inflammatory Markers of Pediatric Acute Appendicitis

**Figure 6:**
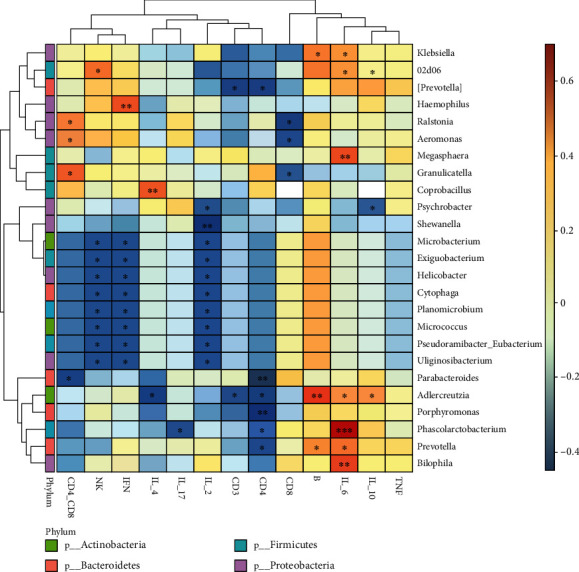
Heatmaps showing correlations between differentially abundant microbiota genera and the production of inflammatory cytokines of pediatric acute appendicitis. IL: interleukin; TNF: tumor necrosis factor; INF: interferon; NK: natural killer; CD4_CD8: CD4/CD8 ratio; B: B cell. Spearman test, ^∗^*P* < 0.05, ^∗∗^*P* < 0.01, and ^∗∗∗^*P* < 0.001.

To explore the effects of gut microflora on inflammatory progression, the correlation analysis between fecal microbiota and inflammatory factors was carried out (Figure 6). *Adlercreutzia* was positively correlated with IL-10 expression (*P* = 0.0419), and the three other genera, including *Klebsiella* (*P* = 0.0279), *Adlercreutzia* (*P* = 0.0027), and *Prevotella* (*P* = 0.035), were positively correlated with B cell infiltration.

## 4. Discussion

Currently, many studies reported that the gut microbiome plays an important role in various pediatric intestinal diseases [15–17]. For example, the recent literature reported that eight groups of microorganisms including Faecalibacterium, an unknown Peptostreptococcaceae, Anaerostipes, Methanobrevibacter, an unknown Christensenellaceae, Collinsella, Fusobacterium, and Escherichia could be used to discriminate Crohn's disease (CD) from non-CD and the six first groups being in lower relative abundance and the last two groups in higher relative abundance in CD [18]. According to Haberman et al. reported, endoscopic appearance of the classic inflammatory process was absent in the ilea of patients with ulcerative colitis (UC) and colon-only CD, while ileal CD-associated gene expression and microbial community differences were remarkably preserved within the colon-only CD subgroup [19]. They also detected an association between depletion of specific Firmicutes and Bacteroidetes taxa and expansion of Proteobacteria and clinical severity [19]. Studies have also shown that digestive tract flora is one environmental factor that induces celiac disease (CeD) [20, 21]. Abdukhakimova et al. summarized the available studies about pediatric CeD and found that Bifidobacterium spp. (e.g., Bifidobacterium longum) was significantly different among all study groups, both in stools and duodenum, in addition to be the only one to significantly correlate between both types of samples [22]. In the study, we will discuss the effects of the gut microflora on appendicitis formation.

The use of antibiotics improves the children's prognosis with appendicitis. Prior studies have not identified the intestinal microbiological differences between appendicitis and health [23–25]. The gut microbial communities of subjects with appendicitis differed from healthy people with antibiotic use [7, 14]. Unlike from the previous studies, we excluded the influence of antibiotic and probiotic use on gut microflora. In the last analysis, Li et al. have pioneered the correlation between gut microbiota and inflammatory factors to explore the diagnostic value of gut microbiota in chronic kidney disease [26]. Our study comprehensively described microbial communities associated with pediatric AA and the production of appendicitis-related inflammatory markers.

A biofilm covers the luminal lining of the large intestinal wall, which prevents pathogens cross the intestinal barrier and aids immune exclusion [27]. Another defense mechanism is the thick and firm mucin layer, which is insoluble to prevent pathogens from damaging intestinal epithelial cells [28]. Furthermore, biofilms were shed actively at regular intervals so that bacteria was left from their surface, facilitating the exclusion of pathogens and the recolonization of beneficial bacteria [27, 29]. However, there is no shedding process in the appendix, which might serve as an area for normal gut microflora and help repopulate normal microbial balance after diarrhea [4, 30, 31]. In this study, the most abundant phyla in both groups were *Firmicutes* and *Bacteroidetes*; however, at the genus level, abundances of *Clostridium* and *Prevotella* increased significantly, and that of Streptococcus reduced considerably in the P group compared to the Y group. *Clostridium* is classically anaerobic rods and ubiquitous in the normal microbial flora of humans, which is an opportunistic pathogen [32]. All pathogenic *Clostridial* species produce protein exotoxins, such as botulinum and tetanus toxins. *Prevotella* is a ubiquitous bacteriopexia and an opportunistic pathogen involved in systemic infection [32]. The enzymes produced by *Prevotella*, such as collagenase and neuraminidase, could play a critical role in pathogenesis. In addition, through analyzing the interaction network among genera, changes in the abundances of particular genera influenced colonization of other genera and further play an important role in the occurrence of pediatric AA, illustrating that alterations in the abundances of gut microflora contribute to appendicitis progression.

The appendix has the highest gut-associated lymphoid tissue (GALT) concentration in the intestine [27]. B cell infiltration increased, and cytokine IL-10 was expressed, thus affecting the immune process [32, 33]. In this study, *Adlercreutzia* was positively correlated with IL-10 expression and *Klebsiella*, *Adlercreutzia*, and *Prevotella* were positively associated with B cell infiltration. Stimulation of B cells by the microbiota can occur by direct interactions, which appear in the context of microbial recognition by surrounding cell types. And considerable indirect communication between the microbiota and B cells is also expected without comma due to changes in bacterial translocation, GALT microenvironment, antigen densities, and T cell responses [34].

Our study has limitations. First, the main limitation of this study is that sample numbers are still relatively limited leading to insufficient power to say anything, for example, about exogenous factors such as environment and dietary habits. To overcome this, we attempted to recruit participants in a small area. However, it cannot be avoided that there is a regional deviation in conclusion. Multicenter studies are required to verify the findings. Moreover, although there is no significant age difference between the two groups in this study and the aged character of the participants is concentrated, the previous studies have shown age-related changes in the microbiome [35, 36]. It is also important to mention that moderate changes of the microbiome can occur over appendicitis development stage. It is essential to mention that the sample size of this study is too small to provide additional comparisons within each age group and categorize different stages of AA. More studies are needed to prospectively assess the alterations of the intestinal microbiome with the child's growth. At last, the study results failed to explicit pathogenicity mechanisms of the microbiome. It still needs to be validated in animal model and clinical investigations.

## 5. Conclusions

In conclusion, the present study's results indicated that significant differences existed in gut microbiome components between pediatric patients with AA and healthy children. Changes in some genera were associated with the production of inflammatory markers in appendicitis. Furthermore, the increase in the abundance of *Adlercreutzia* was positively correlated with IL-10; additionally, changes in gut microbiota might directly stimulate GALT in the appendix and induce an increase of the B cell level.

## Figures and Tables

**Table 1:** Characteristics of the participants in pediatric acute appendicitis group and healthy children group.

Characteristic	Pediatric acute appendicitis group (P group) *n* = 20	Healthy children group (Y group) *n* = 11	*P* value
Age (y)	6.75 ± 2.71	6.00 ± 2.82	0.474
Gender			0.852
Male	12	7	
Female	8	4	
IL-2 (pg/ml)	2.02 ± 1.46	2.00 ± 0.88	0.484
IL-4 (pg/ml)	2.29 ± 2.40	2.04 ± 1.35	0.377
IL-6 (pg/ml)	117.12 ± 257.92	4.43 ± 3.09	0.081
IL-10 (pg/ml)	6.12 ± 6.59	2.63 ± 1.04	0.047^∗^
IL-17 (pg/ml)	7.83 ± 4.54	9.51 ± 5.03	0.175
TNF (pg/ml)	1.59 ± 0.89	1.42 ± 0.73	0.297
IFN (pg/ml)	2.43 ± 3.91	1.38 ± 0.42	0.193
T cells			
CD3^+^ (%)	57.04 ± 11.89	63.48 ± 6.28	0.484
CD4^+^ (%)	24.93 ± 7.58	24.56 ± 5.92	0.445
CD8^+^ (%)	27.81 ± 6.31	32.80 ± 4.56	0.008^∗^
CD4/CD8 ratio	1.19 ± 0.34	1.45 ± 0.58	0.062
NK cell (%)	15.55 ± 9.10	14.88 ± 5.51	0.014^∗^
B cells (%)	25.89 ± 8.77	19.46 ± 3.25	0.013^∗^

Data are shown as the mean SD. IL: interleukin; TNF: tumor necrosis factor; INF: interferon; NK: natural killer. ^∗^*P* < 0.0.

**Table 2:** The distribution at phylum level in pediatric acute appendicitis group and healthy children group.

	P group (%)	Y group (%)
Firmicutes	49.3624	51.4794
Bacteroidetes	33.9665	35.0734
Actinobacteria	8.3718	10.2987
Proteobacteria	6.8897	2.6707
Verrucomicrobia	1.2494	0.3355
Synergistetes	0.000638	00.0192
Euryarchaeota	0.0552	0
Fusobacteria	0	0.0686

## Data Availability

All data used to support the findings of this study are available from the corresponding author upon request.

## Supplementary Materials

Supplementary Materials Supplementary Figure 1: histograms and heatmap at the phylum level in pediatric acute appendicitis group (P group, *n* = 20) and healthy children group (Y group, *n* = 11). Supplementary Figure 2: histograms and heatmap at the top 20 genus level in pediatric acute appendicitis group (P group, *n* = 20) and healthy children group (Y group, *n* = 11). Supplementary Figure 3: box plots of alpha diversity between pediatric acute appendicitis group (P group) and healthy children group (Y group). Supplementary Figure 4: histograms of predictive function analysis of microbial metabolic pathway in pediatric acute appendicitis group (P group, *n* = 20) and healthy children group (Y group, *n* = 11). Supplementary Figure 5: interaction networks among significant genera. Supplementary Table 1: the basic information of the participants of the study. Supplementary Table 2: the proportion of annotations at different levels in pediatric acute appendicitis group and healthy children group. Supplementary Table 3: statistics of alpha diversity.
