# Supplementary material for: The Gut Microbiota and Inflammatory Factors in Pediatric Appendicitis
Source: Dis Markers. 2022 Jul 7;2022:1059445. doi: 10.1155/2022/1059445 (PMC9282992; doi:10.1155/2022/1059445)
Supplement: Supplementary Materials — Supplementary Figure 1: histograms and heatmap at the phylum level in pediatric acute appendicitis group (P group, n = 20) and healthy children group (Y group, n = 11). Supplementary Figure 2: histograms and heatmap at the top 20 genus level in pediatric acute appendicitis group (P group, n = 20) and healthy children group (Y group, n = 11). Supplementary Figure 3: box plots of alpha diversity between pediatric acute appendicitis group (P group) and healthy children group (Y group). Supplementary Figure 4: histograms of predictive function analysis of microbial metabolic pathway in pediatric acute appendicitis group (P group, n = 20) and healthy children group (Y group, n = 11). Supplementary Figure 5: interaction networks among significant genera. Supplementary Table 1: the basic information of the participants of the study. Supplementary Table 2: the proportion of annotations at different levels in pediatric acute appendicitis group and healthy children group. Supplementary Table 3: statistics of alpha diversity. [file 1059445.f1.doc]

Supplementary Material


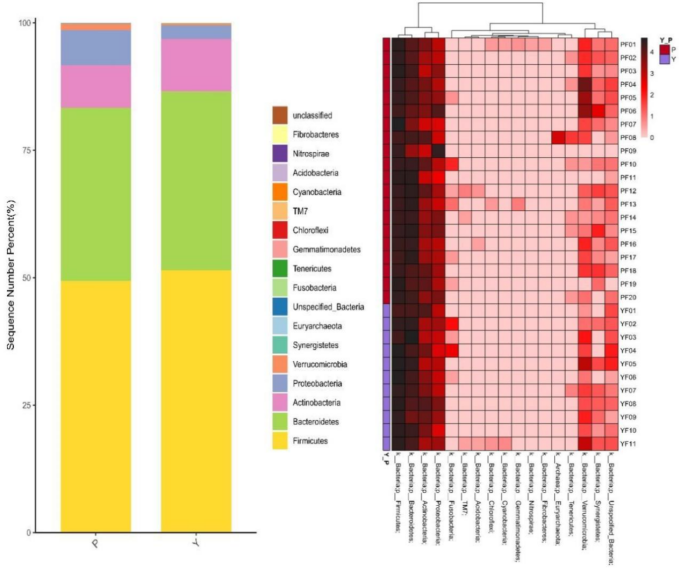


A

B

Supplementary Figure 1 Histograms and heatmap at the phylum level in pediatric acute appendicitis group (P group, n=20) and healthy children group (Y group, n=11).


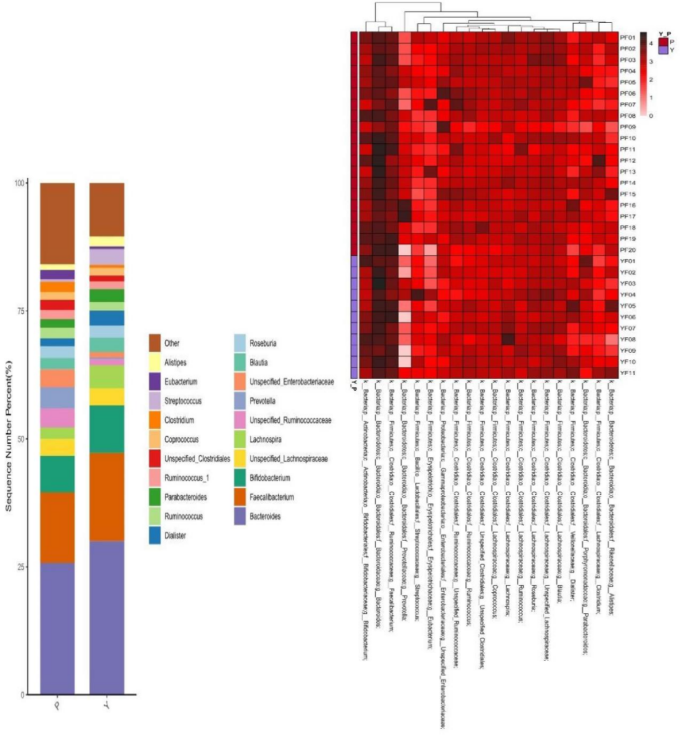


B

A

Supplementary Figure 2 Histograms and heatmapat the Top 20 genus level in pediatric acute appendicitis group (P group, n=20) and healthy children group (Y group, n=11).


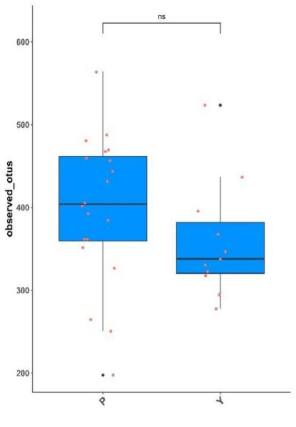

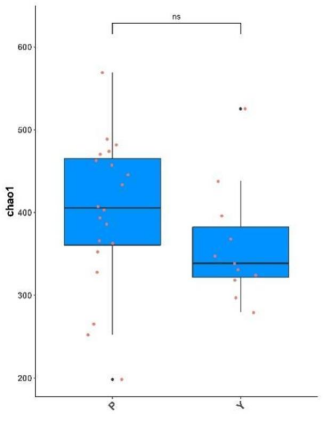


A

B

C

D


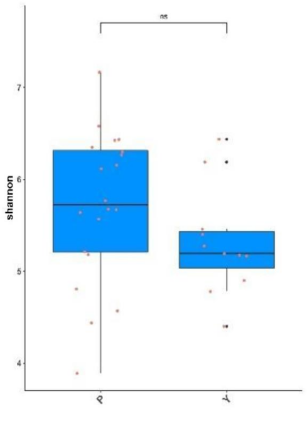

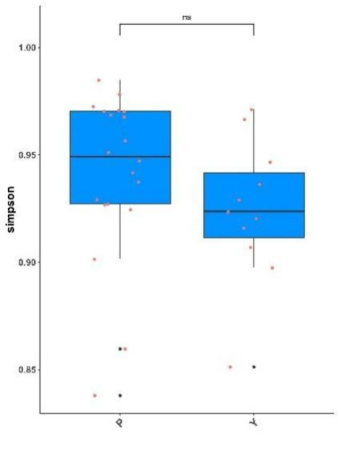


E


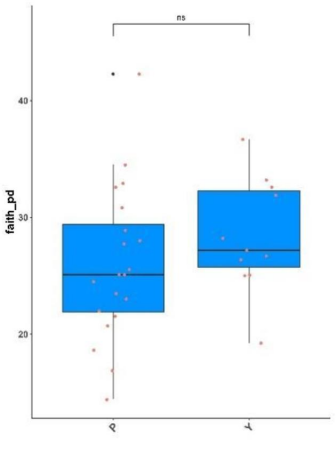


Supplementary Figure 3 Box plots of alpha diversity between pediatric acute appendicitis group (P group) and healthy children group (Y group). ns, no significance. A: Differences in the observed operational taxonomic units between pediatric acute appendicitis group (P group) and healthy children group (Y group); B: Differences in Chao 1 between pediatric acute appendicitis group (P group) and healthy children group (Y group); C: Differences in the Shannon index between pediatric acute appendicitis group (P group) and healthy children group (Y group); D: Differences in the Simpson diversity index between pediatric acute appendicitis group (P group) and healthy children group (Y group). ACE: Index used to estimate the number of OTU in the community; E: Differences in the faith's phylogenetic diversity (faith_pd) index between pediatric acute appendicitis group (P group) and healthy children group (Y group). ns: no significance.


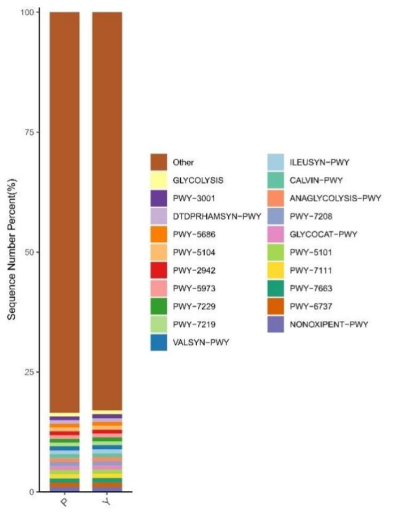


B

A


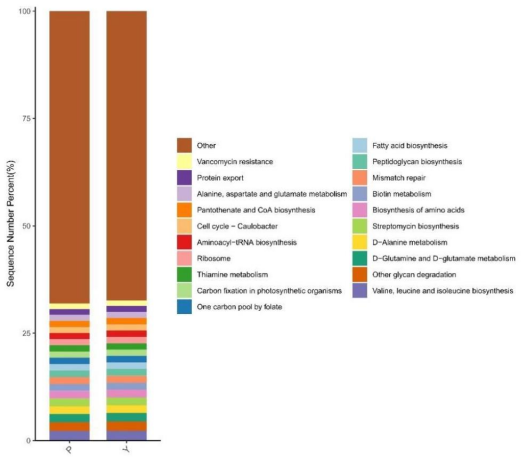


Supplementary Figure 4 Histograms of predictive function analysis of microbial metabolic pathway in pediatric acute appendicitis group (P group, n=20) and healthy children group (Y group, n=11). (A) MetaCycdatabase; (B) KEGG orthology database.


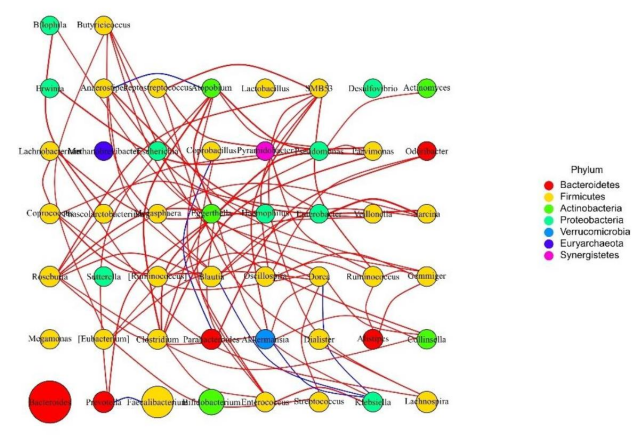


Supplementary Figure 5 Interaction networks among significant genera. The size of each edge presented the Spearman correlation coefficients. Positive correlations were indicated as red edges and inverse correlations as blue edges ((ρ)≥0.5, P < 0.05 for displayed edges).

Supplementary Table 1 The basic information of the participants of the study.

| Sample ID | Gender | Age (y) | IL-2 (pg/ml) | IL-4 (pg/ml) | IL-6 (pg/ml) | IL-10 (pg/ml) | IL-17 (pg/ml) | TNF (pg/ml) |
| --- | --- | --- | --- | --- | --- | --- | --- | --- |
| Y01 | M | 3 | 1.64 | 2.01 | 2.81 | 3.74 | 7.88 | 0.85 |
| Y02 | M | 4 | 2.84 | 1.83 | 10.35 | 3.46 | 10.22 | 1.87 |
| Y03 | F | 4 | 1.81 | 1.15 | 2.06 | 1.66 | 12.56 | 1.38 |
| Y04 | M | 5 | 2.03 | 2.36 | 3.81 | 2.40 | 5.10 | 1.04 |
| Y05 | M | 12 | 1.75 | 1.15 | 3.58 | 2.08 | 10.68 | 1.59 |
| Y06 | M | 8 | 1.47 | 1.40 | 3.73 | 1.97 | 1.47 | 1.04 |
| Y07 | F | 4 | 4.30 | 5.93 | 10.69 | 4.90 | 14.89 | 3.36 |
| Y08 | F | 10 | 2.03 | 1.92 | 2.43 | 3.01 | 1.03 | 1.11 |
| Y09 | M | 5 | 1.53 | 1.15 | 4.21 | 1.50 | 13.49 | 0.59 |
| Y10 | M | 5 | 1.34 | 1.87 | 2.78 | 2.30 | 15.00 | 1.34 |
| Y11 | F | 6 | 1.26 | 1.67 | 2.23 | 1.90 | 12.32 | 1.45 |
| P01 | M | 3 | 1.19 | 0.37 | 7.64 | 10.86 | 0.00 | 0.53 |
| P02 | F | 4 | 1.36 | 1.75 | 39.22 | 6.67 | 6.95 | 1.80 |
| P03 | M | 7 | 2.19 | 0.75 | 28.81 | 2.62 | 7.88 | 1.38 |
| P04 | M | 9 | 0.90 | 3.19 | 1065.37 | 17.31 | 5.56 | 1.73 |
| P05 | F | 3 | 7.01 | 1.57 | 15.10 | 10.37 | 16.76 | 1.45 |
| P06 | M | 13 | 1.75 | 1.40 | 3.81 | 0.84 | 9.75 | 1.18 |
| P07 | M | 10 | 3.38 | 6.14 | 23.75 | 3.23 | 10.22 | 3.90 |
| P08 | M | 8 | 1.86 | 1.15 | 7.39 | 1.29 | 9.28 | 1.38 |
| P09 | F | 6 | 1.81 | 2.18 | 6.89 | 2.62 | 11.62 | 0.91 |
| P10 | M | 4 | 1.53 | 1.83 | 23.22 | 1.81 | 6.95 | 1.45 |
| P11 | M | 10 | 1.64 | 2.45 | 16.41 | 2.73 | 12.09 | 1.87 |
| P12 | F | 8 | 3.76 | 10.89 | 39.22 | 6.20 | 11.15 | 3.82 |
| P13 | F | 3 | 0.00 | 3.10 | 17.63 | 0.00 | 11.62 | 1.59 |
| P14 | M | 8 | 1.70 | 1.40 | 415.38 | 19.71 | 11.15 | 1.52 |
| P15 | M | 5 | 0.96 | 0.99 | 29.34 | 2.95 | 1.92 | 1.04 |
| P16 | F | 7 | 1.53 | 1.32 | 21.82 | 2.40 | 6.95 | 1.04 |
| P17 | M | 5 | 1.86 | 1.15 | 467.72 | 22.40 | 9.75 | 1.87 |
| P18 | M | 6 | 3.16 | 0.00 | 52.74 | 3.46 | 6.95 | 2.08 |
| P19 | F | 9 | 1.42 | 2.10 | 30.47 | 2.40 | 0.00 | 0.66 |
| P20 | F | 7 | 1.42 | 2.10 | 30.47 | 2.40 | 0.00 | 0.66 |

| Sample ID | IFN (pg/ml) | CD3+ (%) | CD4^+^ (%) | CD8^+^ (%) | CD4/CD8 ratio | NK cell (%) | B cells (%) |
| --- | --- | --- | --- | --- | --- | --- | --- |
| Y01 | 0.91 | 61.25 | 23.97 | 30.89 | 0.78 | 12.57 | 22.37 |
| Y02 | 1.45 | 61.15 | 30.87 | 25 | 1.23 | 19.17 | 18.04 |
| Y03 | 1.21 | 66.99 | 32.48 | 29.72 | 1.09 | 8.16 | 22.13 |
| Y04 | 1.03 | 76.16 | 34.9 | 33.77 | 1.03 | 9.07 | 14.18 |
| Y05 | 1.45 | 60.27 | 29.51 | 22.98 | 1.28 | 18.08 | 19.6 |
| Y06 | 0.62 | 67.1 | 39.66 | 21.43 | 1.85 | 13.69 | 16.93 |
| Y07 | 2.14 | 71.24 | 33 | 27.42 | 1.2 | 7.75 | 18.45 |
| Y08 | 1.76 | 58.4 | 33.81 | 21.81 | 1.55 | 13.92 | 26.27 |
| Y09 | 1.52 | 55.76 | 29.55 | 26.09 | 1.13 | 23.32 | 17.08 |
| Y10 | 1.42 | 63 | 40 | 14 | 2.86 | 15 | 20 |
| Y11 | 1.67 | 57 | 33 | 17 | 1.94 | 23 | 19 |
| P01 | 0.62 | 62.3 | 27.03 | 29.57 | 0.91 | 7.82 | 28.33 |
| P02 | 2.53 | 52.44 | 25.34 | 23.31 | 1.09 | 27.1 | 17.24 |
| P03 | 1.64 | 56.49 | 31.75 | 21.86 | 1.45 | 13.3 | 28.97 |
| P04 | 1.27 | 48.37 | 22.61 | 24.24 | 0.93 | 11.41 | 38.48 |
| P05 | 18.61 | 60.76 | 24.07 | 31.14 | 0.77 | 9.33 | 29.11 |
| P06 | 1.33 | 67.08 | 34.26 | 28.24 | 1.21 | 14.87 | 17.05 |
| P07 | 2.59 | 69.4 | 36.13 | 26.33 | 1.37 | 12.8 | 16.49 |
| P08 | 0.97 | 63.02 | 25.91 | 27.53 | 0.94 | 7.71 | 26.68 |
| P09 | 1.39 | 54.92 | 32.67 | 19.9 | 1.64 | 13.66 | 29.96 |
| P10 | 1.89 | 76.05 | 30.49 | 43.79 | 0.7 | 9.94 | 13.48 |
| P11 | 2.40 | 59.96 | 32.91 | 22.38 | 1.47 | 20.07 | 18.02 |
| P12 | 2.27 | 52.98 | 27.6 | 19.14 | 1.44 | 21.78 | 23.72 |
| P13 | 0.16 | 48.01 | 17.92 | 22.29 | 0.8 | 5.7 | 44.41 |
| P14 | 3.86 | 29.45 | 15.61 | 9.16 | 1.7 | 22.48 | 46.1 |
| P15 | 1.45 | 68.17 | 31.06 | 33.86 | 0.92 | 9.99 | 21.15 |
| P16 | 1.76 | 65.72 | 37.16 | 23.9 | 1.56 | 9.11 | 23.19 |
| P17 | 1.52 | 33.12 | 19.64 | 10.97 | 1.79 | 41.36 | 24.23 |
| P18 | 0.00 | 44.58 | 19.53 | 22.21 | 0.88 | 30.74 | 23.52 |
| P19 | 1.21 | 64 | 32.33 | 29.41 | 1.1 | 10.93 | 23.82 |
| P20 | 1.21 | 64 | 32.33 | 29.41 | 1.1 | 10.93 | 23.82 |

Supplementary Table 2 The proportion of annotations at different levels in pediatric acute appendicitis group and healthy children group.

| **Groups** | **Kingdom** | **Phylum** | **Class** | **Order** | **Family** | **Genus** | **Species** |
| --- | --- | --- | --- | --- | --- | --- | --- |
| P group | 1 | 0.99978 | 0.99976 | 0.99973 | 0.97983 | 0.85654 | 0.45368 |
| Y group | 1 | 0.99949 | 0.99949 | 0.99947 | 0.98798 | 0.92685 | 0.43620 |

Supplementary Table 3 Statistics of alpha diversity.

| **α** | **acute appendicitis group (P group)** | **healthy children group (Y group)** | **P value** |
| --- | --- | --- | --- |
| chao1 | 400.2786±90.301 | 360.6271±70.5083 | 0.2186 |
| shannon | 5.7133±0.8253 | 5.3100±0.5831 | 0.1630 |
| Simpson | 0.9413±0.0386 | 0.9242±0.0333 | 0.2264 |
| faith_pd | 25.9589±6.5822 | 28.4031±4.8647 | 0.2903 |
| observed_otus | 389.35±89.6028 | 359.5455±70.5399 | 0.3497 |
